# The associations of early and surgical menopause with 10-year employment trajectories bracketing final menstruation or surgery

**DOI:** 10.1097/GME.0000000000002640

**Published:** 2025-10-07

**Authors:** Darina Peycheva, Bożena Wielgoszewska, Paola Zaninotto, Andrew Steptoe, Rebecca Hardy

**Affiliations:** 1 UCL Institute of Epidemiology and Health Care, London, UK; 2 UCL Social Research Institute, University College London, UK; 3 School of Sport, Exercise and Health Sciences, Loughborough University, Epinal Way, Loughborough, Leicestershire, UK

**Keywords:** Early menopause, Employment trajectories, English Longitudinal Study of Aging, Menopause policy, Postmenopause hormone therapy, Premenopausal bilateral oophorectomy, Premenopausal hysterectomy, Surgical menopause

## Abstract

**Objectives:**

This study examines the employment trajectories of women experiencing early and surgical menopause over a 10-year period bracketing their final menstruation or surgery, representing for most women the menopause transition. It also investigates the potential mediating role of hormone therapy in early postmenopause in these relationships.

**Methods:**

We used data from 1,386 women in the English Longitudinal Study of Aging (ELSA) who had undergone natural menopause, premenopausal bilateral oophorectomy or hysterectomy. We used sequence analysis of employment histories to define 3 different 10-year employment trajectories. We then carried out regression analysis to assess associations between timing and type of menopause on employment, followed by mediation analysis. Sensitivity analysis was conducted by excluding cases with hysterectomy with preserved ovaries.

**Results:**

Women with early menopause, compared with those who undergo menopause at 45 or older, are less likely to have flexible working arrangements (part-time work or self-employment) compared with full-time work during this sensitive period (relative risk ratio [RRR], 0.70; 95% CI: 0.51-0.97). However, the likelihood of leaving the labor market compared with working full-time is similar in women with early and later menopause (RRR, 0.95; 95% CI: 0.62-1.41). Surgical menopause, compared with natural menopause, is associated with an increased risk of labor market exit (RRR, 1.45; 95% CI: 1.01-2.32), particularly for women aged 45 or older at the time of surgery (RRR, 1.50; 95% CI: 0.94-2.38). Hormone therapy use may help reduce the risk of labor market exit for women with both early (RRR_NATURAL INDIRECT EFFECT [NIE]_, 0.79; 95% CI_BIAS-CORRECTED [BC]_, 0.58-1.04) and surgical menopause (RRR_NIE_, 0.73; 95% CI_BC_, 0.53-1.01). Sensitivity analysis suggests that the potential reduction in labor market exit risk via hormone therapy for early menopausal women holds true only when women with hysterectomy with preserved ovaries are included.

**Conclusions:**

Our study highlights that early menopause and surgical menopause, including hysterectomy with preserved ovaries, impact women’s labor market trajectories and suggests that hormone therapy within the early years of the final menstruation may help women remain employed. We advocate for further research on the impact of the timing and type of menopause on women’s labor market circumstances and for workplace policies that consider their diverse experiences.

Age of menopause is widely regarded as a key marker of women’s health.^1^ Notably, menopause before the age of 45, also known as early menopause, is associated with an increased risk of cardiovascular disease, osteoporosis, and premature mortality.^2^ Women with early menopause are also at higher risk of experiencing more frequent, severe, and prolonged hot flushes and night sweats, as well as depressive symptoms.^3^ Premature morbidity and symptomatic menopause can be even more pronounced in women who undergo early surgical menopause or premenopausal hysterectomy, where ovarian function ceases abruptly or menopause occurs earlier due to disrupted ovarian blood flow.^4-6^

Despite extensive research on the health consequences of early and surgical menopause, there is limited evidence on their impact on other aspects of women’s lives, including labor market experiences. Many women go through menopause while at work, and their experiences may influence their employment. Previous studies on menopause and women’s labor market circumstances have primarily focused on menopausal symptoms.^7,8^ Similarly, workplace policies have largely addressed symptom management.^9,10^ To our knowledge, few studies have investigated the role of earlier menopause on work.^11-14^ Quantitative studies from Britain and Scandinavia have generally shown early natural menopause to be associated with lower work participation^12-14^; however, these studies did not consider surgery and did not consider the impact of hormone therapy (HT) as a mediator. HT is often used by women who undergo early natural menopause or surgery (bilateral oophorectomy and hysterectomy), and for the treatment of menopause symptoms.^15,16^ Therefore, HT use could be beneficial in allowing women to maintain their employment participation. Understanding how menopause timing and type affect employment outcomes, and the role of HT, could be essential for shaping effective policies.

Here, we examine the different employment trajectories around the menopause transition and how these are associated with early menopause (before 45 years) and surgical menopause (premenopausal bilateral oophorectomy or hysterectomy). In addition, we explore the potential mediating role of HT use in early postmenopause in the relationship between early and surgical menopause and employment trajectories.

## METHODS

### Study population

We used data from the English Longitudinal Study of Aging (ELSA), a representative cohort of men and women aged 50 and older in England, which provides a multidisciplinary resource for research and policy on aging. Specifically, we used data from the first Life History interview, a substudy of ELSA^17^ administered in 2007,^18,19^ which collected retrospective data on participants’ earlier life experiences, including employment histories.

In total, 4,779 women core study members were eligible for the Life History interview. A total of 3,912 participated in the interview. Our analytical sample consists of 1,386 women who had reached the state pension age of 60 in these cohorts^20^ and had undergone either natural menopause or premenopausal bilateral oophorectomy or hysterectomy. We excluded women who experienced late menopause or surgery (after 55 years) due to their limited employment time before state pension age. We also excluded women not employed at the start of the observation period and those with missing employment history data in this period. This allows us to observe employment changes related to menopause. A detailed flow diagram outlining the inclusion and exclusion criteria is presented in Supplemental Figure S1, Supplemental Digital Content 1, http://links.lww.com/MENO/B409.

### Outcomes

We used employment history data from the ELSA 2007 Life History interview, which included information on economic activity, such as paid jobs lasting 6 months or longer and periods of nonemployment of 3 months or more, with associated start and end dates. Due to small proportions in some economic activities, we analyzed work status information across three categories: full-time employment, flexible working arrangements (including part-time work and self-employment), and not in paid employment (including unemployment, home/family work, retirement, and other nonemployment states).

We focused on a 10-year period bracketing the final menstrual period (FMP), 5 years leading up to and 5 years following the FMP (with the FMP itself included in the latter period). This time frame typically corresponds to late perimenopause and early postmenopause.^21^ For women who have undergone bilateral oophorectomy or hysterectomy, we used the date of surgery as the reference point.

We used sequence analysis to identify employment trajectories, capturing patterns in how women transition through different states over time. The FMP serves as the time frame midpoint but does not directly determine clustering. We employed optimal matching (OM) to quantify sequence similarity or dissimilarity.^22^ Using the computed distance matrix, we performed agglomerative hierarchical clustering to group similar sequences. We also explored alternatives like Partitioning Around Medoids (PAM) and combined PAM and hierarchical clustering, considering various group sizes. Our evaluation criteria included clustering quality measures and theoretically relevant solutions.^23^

We found an eight-cluster solution most informative, which we combined into three conceptually similar groups for analysis (Table 1). These were (1) continuous full-time employment or transition to full-time employment (cluster 1), (2) continuous part-time employment, self-employment, or transition to part-time or self-employment (cluster 2), and (3) transitions out of the labor market (unemployment, retirement, looking after home or family, and other nonemployed statuses) (cluster 3) (Supplemental Figure S2, Supplemental Digital Content 1, http://links.lww.com/MENO/B409).

**TABLE 1:** Clusters of employment trajectories bracketing the final menstruation, n=1,386

Description	n (%)*a*	Dominant states (%)
Cluster 1: Continuous full-time employment, or transition to full-time employment	561 (39.6)	
Continuous FTE	503 (35.6)	FTE (98.9)
Transition from PTE or SE to FTE	58 (4.0)	FTE (57.3); PTE or SE (33.4)
Cluster 2: Continuous part-time employment, self-employment, or transition to part-time or self-employment	600 (44.5)	
Continuous PTE or SE	548 (41.1)	PTE or SE (96.6)
Transition from FTE to PTE or SE	52 (3.5)	FTE (51.1); PTE or SE (46.5)
Cluster 3: Transitions out of the labor market (unemployment, retirement, looking after home or family, other nonemployed statuses)	225 (15.9)	
Transition from PTE or SE to NE	51 (3.5)	PTE or SE (50.7); NE (45.6)
Transition from PTE or SE to NE	41 (3.0)	PTE or SE (20.2); NE (77.7)
Transition from FTE to NE	74 (5.1)	FTE (66.3); NE (33.5)
Transition from FTE to NE	59 (4.3)	FTE (26.8); NE (64.3)

FTE, full-time employment; n, number; NE, not in employment; PTE, part-time employment; SE, self-employment.

^*a*^ Unweighted n (weighted %).

### Exposures and mediators

In the ELSA 2007 Life History interview, women were asked about the year of their FMP and the reason for amenorrhea if they had not experienced a menstrual period in the 12 months preceding the interview. In addition, they were asked about their history of hysterectomy and bilateral oophorectomy, along with the years of these surgeries. Information on HT use was collected, including details about the start and end years.

Early menopause was defined as having experienced natural menopause or premenopausal bilateral oophorectomy or hysterectomy before age 45. Due to small sample sizes, women who underwent bilateral oophorectomy or hysterectomy before their FMP were grouped into a single “surgical menopause” category. Although hysterectomy with intact ovaries does not lead to immediate menopause, it can lead to an earlier onset due to reduced ovarian blood supply. In addition, hysterectomy surgery itself may influence labor market participation. Women without a menstrual period in the 12 months preceding the interview and no obvious physiological reasons for the amenorrhea were defined as having natural menopause.

Women who started HT within 5 years of their FMP or surgery were categorized as using HT in the early years of postmenopause. This included women already on HT before surgery, as it is advised to continue HT after surgery.^24^ Women who initiated HT after 5 years of FMP or never used it were classified as having no HT use.

We excluded women who were on HT before their FMP and reported no obvious reason for amenorrhea, because it was not possible to assign an accurate date of menopause. We also excluded women with other reasons for the absence of menstruation (such as chemotherapy, contraceptive, or other medications) or whose menopause status could not be determined due to insufficient information.

### Covariates

This analysis accounts for a range of additional measures preceding the 10-year study time frame, which are known to influence both the experience of menopause and employment opportunities and trajectories.^25,26^ Childhood characteristics included were adverse childhood experiences (ACEs) (no ACEs vs. any ACEs), self-rated childhood health (excellent or good vs. fair, poor, or variable), and childhood smoking (no vs. yes). Childhood is defined as “before age 16”. ACEs included experiences of parental death or separation, parental substance misuse, parental conflict, parental abuse, or parental unemployment lasting longer than 6 months, and age at menarche (aged 12 or older vs. aged 11 or younger (early menarche). Adulthood characteristics included were education (some education vs. no educational qualifications), previous labor market participation (proportion of time spent working from age 15, end of compulsory school age, until the onset of the period of observation), and nulliparity (no live born children vs. live born children).

In addition, we included whether women had children under 18 (no vs. yes), poor health (no vs. yes), were living with a partner (no vs. yes), and their accommodation type (owned vs. renter or other) at the start of the 10-year analysis period. We also accounted for age (in years) at the 2007 Life History interview and generation (born before 1928, born 1928-1945, and born 1946-1964).

### Statistical analysis

We employed multinomial logistic regression to estimate relative risk ratios for being continuously in, or transition to, part-time or self-employment (cluster 2) or exiting the labor market (cluster 3) relative to being continuously in, or transitioning to, full-time employment (cluster 1), for women with early versus nonearly menopause and surgical versus natural menopause. The relative risk ratios can also be interpreted as conditional odds ratios,^27^ representing the odds of being in cluster 2 or 3 compared with cluster 1. First, we calculated relative risk ratios in separate models for early and surgical menopause, followed by a model including an interaction term between the two. We used staged covariate adjustment, first accounting for age and generation (model 1), then adding childhood characteristics (model 2), and finally including all covariates (model 3). We used survey weights to correct for sampling probabilities and differential nonresponse.^19^

We quantified the mediating role of postmenopause HT using causal mediator analysis with inverse odds weighting (IOW).^28,29^ The odds were obtained using a logistic regression model for the exposure given the mediator and confounding variables, accounting for sampling probabilities and differential nonresponse. The weight was computed by taking the inverse of the predicted odds for each observation in the exposed group; the unexposed group was assigned an IOW of 1. The total effect (TE) of the exposure was estimated from multinomial logistic regression model of the outcome on exposure and confounding variables, accounting for sampling probabilities and differential nonresponse. The natural direct effect (NDE) of the exposure was estimated via multinomial logistic regression model of the outcome on exposure and confounding variables, using the IOW multiplied by the survey weight. The natural indirect effect (NIE) via the mediator was estimated by subtracting the NDE from the TE and using 10,000 bootstrap replications to derive bias-corrected CIs for TE, NDE, and NIE.

In secondary analysis, we replicated the study after excluding women who had premenopausal hysterectomies with preserved ovaries (n=194), restricting the surgical menopause group to cases induced through bilateral oophorectomy (n=160). Due to small group sizes, we were not able to analyze the joint associations between early and surgical menopause.

The sequence and cluster analyses were conducted using R version 4.2.2. Descriptive statistics and regression modeling were conducted using Stata 18.

We used multiple imputation (MI) with chained equations with 20 data sets to address missing data in the covariates. The imputation model includes the outcomes, exposures, mediator, and covariates.^30^ The proportion of missing observations for each variable is shown in Table 2.

**TABLE 2:** Sample characteristics (mean/percentages) by clusters of employment trajectories bracketing the final menstrual period, n=1,386

Characteristics	n (%)*a* complete case	% imputed sample	Employment trajectories bracketing the final menstrual period			
			Cluster 1: continuous FTE or transition to FTE (n=561)	Cluster 2: continuous PTE, SE, or transition to PTE or SE (n=600)	Cluster 3: transitions out of the labor market (n=225)	*P* *b*
Early menopause						
No	1,081 (77.7)		73.7	82.0	76.1	0.002
Yes	305 (22.3)		26.3	18.0	23.9	
Surgical menopause						
No	1,032 (74.5)		75.9	75.1	69.6	0.160
Yes	354 (25.5)		24.1	24.9	30.4	
Menopause type and time						
Not early natural menopause	892 (64.2)		61.5	68.8	58.7	<0.001
Not early surgical menopause	189 (13.5)		12.2	13.2	17.4	
Early natural menopause	140 (10.3)		14.5	6.03	10.9	
Early surgical menopause	165 (12.0)		11.8	11.7	13.0	
Age						
Mean (SD)	72.0 (8.3)		73.0 (8.6)	71.3 (8.1)	71.2 (7.7)	0.001
Median (range)	71 (60-92)		72 (60-92)	70 (60-92)	70 (60-92)	
Generation						
Born before 1928	262 (19.9)		24.6	17.9	13.5	0.002
Born 1928-1945	970 (69.1)		64.7	70.4	76.5	
Born 1946-1964	154 (11.0)		10.7	11.7	10.0	
Adverse childhood experiences						
No	974 (70.3)		69.4	71.7	68.7	0.573
Yes	412 (29.7)		30.6	28.3	31.3	
Childhood smoking						
No	1,240 (89.4)	89.7	89.6	90.0	89.1	0.984
Yes	141 (10.3)	10.3	10.4	10.0	10.9	
mi	5 (0.3)					
Self-rated childhood health						
Excellent or good	1,223 (88.1)	88.1	90.0	86.0	89.1	0.084
Fair, poor, or variable	162 (11.9)	11.9	10.0	14.0	10.9	
mi	1 (0.1)					
Menarche						
aged 12 or older	1,077 (77.9)	78.0	77.2	78.5	78.7	0.639
aged 11 or younger	307 (22.0)	22.0	22.8	21.5	21.3	
mi	2 (0.1)					
Education						
Some education	847 (60.2)		63.7	58.7	55.7	0.063
No educational qualification	539 (39.8)		36.3	41.3	44.4	
Previous labor market participation (proportion of time spent working)						
Mean (SD)	0.69 (0.23)	0.68 (0.23)	0.71 (0.23)	0.67 (0.22)	0.65 (0.25)	<0.001
Median (range)	0.69 (0-1)	0.69 (0-1)	0.71 (0-1)	0.67 (0.09-1)	0.66 (0-1)	
mi	30 (2.2)					
Nulliparous						
No (live-born biological children)	1,146 (82.7)		75.2	90.0	81.7	<0.001
Yes (no live-born biological children)	240 (17.3)		24.8	10.0	18.3	
Children under 18 at the start of the period of observation						
No	620 (44.5)		47.5	37.6	55.7	<0.001
Yes	766 (55.5)		52.5	62.4	44.4	
Poor health at the start of the period of observation						
No	1,242 (89.9)	89.9	90.5	91.5	84.3	0.009
Yes	143 (10.1)	10.1	9.5	8.5	15.7	
mi	1 (0.1)					
Living with a partner at the start of the period of observation						
No	248 (18.1)		25.1	8.4	27.0	<0.001
Yes	1,138 (81.9)		74.9	91.6	73.0	
Living in owned property at the start of the period of observation						
No (rented or other)	451 (33.6)		39.1	28.1	34.4	<0.001
Yes (owned)	935 (66.4)		60.9	71.9	65.7	
Post menopause HT						
Within 5 years of FMP or surgery						
No	1,076 (78.2)	79.4	80.5	79.5	76.3	0.399
Yes	290 (20.4)	20.6	19.5	20.5	23.7	
mi	20 (1.4)					

HT, hormone therapy; FMP, final menstrual period; FTE, full-time employment; mi, missing; n, number; PTE, part-time employment; SE, self-employment.

^*a*^ Unweighted n (weighted %).

^*b*^ *P*-values refer to the relevant statistical test (χ^2^ test, F-test).

## RESULTS

Cluster 1 (39.6%) comprised women predominantly engaged in full-time employment or transitioned to full-time employment (Table 1). Cluster 2 (44.5%) included those working part-time, being self-employed, or transitioning to part-time employment or self-employment. Cluster 3 (15.9%) consisted of women who exited the workforce, including those who were unemployed, caring for home or family, or engaged in other forms of non-employment.

Table 2 presents the characteristics of the sample of 1,386 women aged 60 to 92 years (born between 1915 and 1947), with a mean age at interview of 72 years. Of these, 22.3% experienced menopause before 45 years: 10.3% experienced early natural menopause and 12.0% early surgical menopause. In total, 25.5% underwent surgical menopause before their final menstrual period. 20.6% used HT within the 5 years following the final menstrual period or surgery.

Women in cluster 1 had the highest percentage of early menopause and cluster 2 the lowest, but cluster 3 had the highest percentage experiencing surgical menopause (Table 2). Women in cluster 1 were older at interview, most likely to have some education, to have no biological children and to have previously spent a larger proportion of time in the labor market. Those in cluster 2 were most likely to have biological children, to be living with a partner and to be living in an owned property at the start of the observation. Women in cluster 3 were least likely to have some education, most likely to have reported ill health, and least likely to have children under 18 at the start of the observation period.

Table 3 presents the separate effects of early menopause and surgical menopause on employment trajectories. In fully adjusted models (model 3), compared with women in full time employment (cluster 1), those experiencing early menopause (relative to later menopause) were 30% less likely to work part-time or be self-employed (cluster 2) (RRR, 0.70; 95% CI: 0.51-0.97). However, the likelihood of leaving the labor market (cluster 3), as opposed to working full-time (cluster 1), did not differ between women with early and later menopause. Women with experience of surgical menopause (compared with those with natural menopause) were more likely to exit the labor market than be in full-time employment (RRR, 1.45; 95% CI: 0.99-2.11). Full details are presented in Supplemental Table S1, Supplemental Digital Content 1, http://links.lww.com/MENO/B409.

**TABLE 3:** Relative risk ratios (RRRs) and 95% CIs of early and surgical menopause and their association with clusters of employment trajectories, n=1,386*a*

	Employment trajectories bracketing the final menstrual period	
	RRR (95% CI)	
Menopause characteristics	Cluster 2: Continuous PTE, SE, or transition to PTE or SE (reference: cluster 1: continuous FTE or transition to FTE)	Cluster 3: Transitions out of the labor market (reference: cluster 1: continuous FTE or transition to FTE)
Early menopause (ref: no)*b*		
Model 1	0.62 (0.46-0.84)	0.86 (0.59-1.26)
Model 2	0.62 (0.46-0.85)	0.87 (0.59-1.27)
Model 3	0.70 (0.51-0.97)	0.95 (0.63-1.43)
Surgical menopause (ref: no)*b*		
Model 1	1.03 (0.77-1.36)	1.28 (0.90-1.83)
Model 2	1.02 (0.77-1.36)	1.29 (0.90-1.85)
Model 3	1.05 (0.78-1.42)	1.45 (0.99-2.11)

FTE, full-time employment; n, number; PTE, part-time employment; RRR, relative risk ratio; SE, self-employment.

^*a*^ Imputed weighted data.

^*b*^ Model 1 is adjusted for age and cohort, model 2 is further adjusted for childhood factors: adverse childhood experiences, self-rated childhood health, childhood smoking, and menarche, and model 3 is further adjusted for adulthood factors: education, previous labor market participation, nulliparity, children under 18, illness, cohabiting partner, and accommodation type. Full details are presented in Supplemental Table S1, Supplemental Digital Content 1, http://links.lww.com/MENO/B409.

Table 4 presents the combined effect of early and surgical menopause. In fully adjusted models, women with early natural menopause (compared with later natural menopause) were less likely to work part-time than to work full-time (RRR, 0.47; 95% CI: 0.29-0.76). Women with both early and later surgical menopause had an increased risk of leaving the labor market compared with later natural menopause, with the greatest risk observed in those with surgical menopause at 45 or older (RRR, 1.50; 95% CI: 0.94-2.38). Full details are presented in Supplemental Table S2, Supplemental Digital Content 1, http://links.lww.com/MENO/B409.

**TABLE 4:** Relative risk ratios (RRRs) and 95% CIs of the combination of early and surgical menopause and their association with clusters of employment trajectories, n=1,386*a*

	Employment trajectories bracketing the final menstrual period	
	RRR (95% CI)	
Menopause characteristics	Cluster 2: continuous PTE, SE, or transition to PTE or SE (reference: cluster 1: Continuous FTE or transition to FTE)	Cluster 3: transitions out of the labor market (reference: cluster 1: continuous FTE or transition to FTE)
Model 1 (ref. natural menopause at 45 or older)*b*		
Surgical menopause at 45 or older	0.97 (0.67-1.40)	1.45 (0.92-2.27)
Early natural menopause	0.40 (0.25-0.62)	0.82 (0.48-1.39)
Early surgical menopause	0.87 (0.59-1.28)	1.05 (0.64-1.72)
Model 2 (ref. natural menopause at 45 or older)*b*		
Surgical menopause at 45 or older	0.97 (0.67-1.40)	1.45 (0.92-2.28)
Early natural menopause	0.40 (0.26-0.63)	0.83 (0.49-1.40)
Early surgical menopause	0.87 (0.58-1.28)	1.06 (0.64-1.75)
Model 3 (ref. natural menopause at 45 or older)*b*		
Surgical menopause at 45 or older	0.95 (0.64-1.39)	1.50 (0.94-2.38)
Early natural menopause	0.47 (0.29-0.76)	0.83 (0.49-1.41)
Early surgical menopause	0.95 (0.63-1.43)	1.28 (0.75-2.20)

FTE, full-time employment; n, number; PTE, part-time employment; RRR, relative risk ratio; SE, self-employment.

^*a*^ Imputed weighted data.

^*b*^ Model 1 is adjusted for age and cohort, model 2 is further adjusted for childhood factors: adverse childhood experiences, self-rated childhood health, childhood smoking, and menarche, and model 3 is further adjusted for adulthood factors: education, previous labor market participation, nulliparity, children under 18, illness, cohabiting partner, and accommodation type. Full details are presented in Supplemental Table S2, Supplemental Digital Content 1, http://links.lww.com/MENO/B409.

Surgical menopause was strongly associated with postmenopausal HT use (RRR, 4.83; 95% CI, 3.48-6.71), though there was no clear evidence of an association between early menopause and HT use, or between HT use and employment trajectory (Supplemental Table S3, Supplemental Digital Content 1, http://links.lww.com/MENO/B409).

Table 5 displays the causal effect estimates from the decomposition of the total effect of early menopause and surgical menopause on employment trajectory. There was no evidence of mediation by postmenopausal HT use (RRR_NIE,_ 1.05; 95% CI_BC,_ 0.88-1.26) of the association between early menopause and being less likely to be in part-time employment compared with full-time employment. The difference between the TE and NDE estimates was small. Despite no evidence of an association between early menopause and transitions out of the labor market, there was a suggestion that the use of HT could mediate this relationship (RRR_NIE_, 0.79; 95% CI_BC_, 0.58-1.04) (known as inconsistent mediation; the NIE and NDE have different directions, offsetting rather than augmenting each other). This suggests that if all women with early menopause had used HT during the study period, the likelihood of leaving work rather than remaining in full-time employment could have been reduced. There was some evidence that HT use mediated the relationship between surgical menopause and an increased likelihood of exiting the labor market compared with working full-time (RRR_NIE_, 0.73; 95% CI_BC_, 0.53-1.01). However, other factors could also play a role in mediating this association. Independent of HT use, premenopausal surgery was associated with a higher likelihood of labor market exit, compared with full-time employment (RRR_NDE_, 1.98; 95% CI_BC_, 1.16-3.31).

**TABLE 5:** Mediation of the association between early and surgical menopause and clusters of employment trajectories by postmenopause hormone therapy, n=1,386*a*

	Employment trajectories bracketing the final menstrual period	
	RRR (95% CI)	
Menopause characteristics Effects	Cluster 2: continuous PTE, SE, or transition to PTE or SE (reference: cluster 1: continuous FTE or transition to FTE)	Cluster 3: Transitions out of the labor market (reference: cluster 1: continuous FTE or transition to FTE)
Early menopause (ref: no)*b* *c*		
Total effect	0.70 (0.51-0.98)	0.95 (0.62-1.45)
Indirect effect (acting through HT)	1.05 (0.87-1.27)	0.79 (0.58-1.04)
Direct effect (unexplained by HT)	0.67 (0.47-0.96)	1.20 (0.74-1.96)
Surgical menopause (ref: no)*b* *c*		
Total effect	1.05 (0.77-1.43)	1.45 (0.98-2.12)
Indirect effect (acting through HT)	0.90 (0.69-1.16)	0.73 (0.53-1.01)
Direct effect (unexplained by HT)	1.17 (0.79-1.74)	1.98 (1.16-3.31)

^*a*^ Imputed weighted data.

^*b*^ Model is adjusted for age and cohort, childhood factors: adverse childhood experiences, self-rated childhood health, childhood smoking, and menarche, and adulthood factors: education, previous labor market participation, nulliparity, children under 18, illness, cohabiting partner, and accommodation type.

^*c*^ 95% CI_BC_ based on 10,000 bootstrap replications.

BC, bias-corrected; FTE, full-time employment; HT, hormone therapy; n, number; PTE, part-time employment; RRR, relative risk ratio; SE, self-employment.

In secondary analysis (Supplemental Table S7, Supplemental Digital Content 1, http://links.lww.com/MENO/B409), excluding women who had premenopausal hysterectomies with preserved ovaries, women with early menopause (compared with later menopause) remained less likely to work part-time than full-time (RRR_TE_, 0.60, 95% CI_BC_, 0.39-0.93). Consistent with the primary analysis, there was no evidence linking early menopause to leaving the labor market (RRR_TE_, 0.99; 95% CI_BC_, 0.63-1.54). However, the previously observed potential protective effect of HT was no longer evident (RRR_NIE_, 1.01; 95% CI_BC_, 0.70-1.44). Women who experienced surgical menopause (compared with natural menopause) remained at an increased risk of leaving the labor market with similar RRR (RRR_TE_, 1.43; 95% CI_BC_, 0.88-2.30). HT could have helped mitigate this risk (RRR_NIE_, 0.64; 95% CI_BC_, 0.40-0.98). As in the primary analysis, the estimated direct effect remained large (RRR_NDE_, 2.24; 95% CI_BC_, 1.15-4.18). Further details of this analysis are provided in Supplemental Tables S4-7, Supplemental Digital Content 1, http://links.lww.com/MENO/B409 and Supplemental Figure S3, Supplemental Digital Content 1, http://links.lww.com/MENO/B409.

## DISCUSSION

Compared with women with later menopause, women with early menopause were less likely to work in flexible arrangements (such as part-time or self-employed) and more likely to work standard full-time jobs. However, regardless of the age of menopause onset, women were equally likely to leave the labor market as to work full-time. Women who underwent premenopausal bilateral oophorectomy or hysterectomy had a higher risk of leaving the labor market than working full-time compared with those undergoing natural menopause, particularly if surgery occurred after age 45 years. HT use in the early years of the final menstruation may support women with early and surgical menopause, including hysterectomy with preserved ovaries, in maintaining full-time employment rather than exiting the workforce.

Our finding that women with early menopause spent more time in full-time employment compared with more flexible forms of employment may imply that these women exert extra effort to maintain work stability. Previous studies have shown that menopausal women, compared to premenopausal and perimenopausal women, are more likely to be working and have a lower intention to leave the labor market.^31,32^ Research has also highlighted menopausal women’s resilience and determination to conceal vulnerabilities related to menopause.^33,34^ Our evidence shows that this may particularly be the case among women with early menopause. Their effort could be influenced by the stigma associated with undergoing the experience at an earlier age than usual and the financial need to keep working at this younger age. It is also possible that the menopause transition does not significantly disrupt labor market participation, at least in the short term, depending on factors such as sociodemographic and work characteristics. Previous research has noted that supportive workplaces were associated with intact career trajectories among educated women with early menopause, which was not the case for less advantaged women.^11^ In a Scandinavian study, menopause complaints before the age of 45 (as a marker of early menopause) did not significantly impact the likelihood of working full-time in the short term, but 3 to 4 years after the initial menopause-related complaint, there was a reduction in labor supply.^12^ Our findings partly align with a previous British study in a cohort born in 1958 that early menopause was linked to subsequent reduced overall employment rates, but not full-time employment rates.^14^ These differences in the effects on full-time employment could be attributed to generational differences, as the majority included in our study were born before 1958.

Our finding that premenopausal bilateral oophorectomy or hysterectomy at any age is a risk factor for labor market exit is concerning, not only because of the precariousness that gynecological surgeries pose for women’s employment, but also because of the number of women who undergo such surgeries. While more support is now available for women undergoing these surgeries than in the past,^35^ continued efforts are needed to better understand and normalize their experiences and to mitigate the impact on their employment. To our knowledge, this study is the first to quantify the independent effect of surgical menopause, including hysterectomy with preserved ovaries, on employment trajectories during the period surrounding the surgery. A qualitative study has noted that beyond symptoms, the effects of early or premature surgical menopause and complex treatments contributed to interrupted or altered careers.^11^ Other research focused on the health consequences of bilateral oophorectomy and hysterectomy,^4,36^ which might be an important pathway to employment disruption. It is thus not surprising that women 45 or older with experience of gynecological surgery may be even more affected compared with those who had the surgery at a younger age due to higher comorbidities and increased precarity related to age.

The finding that HT use in the early years of the final menstruation may enable women to work full-time instead of leaving the labor force aligns with previous research suggesting that HT has a beneficial effect on menopausal experiences and could therefore minimize adverse work outcomes.^33,37^ However, this may particularly be the case among women with surgical menopause, including hysterectomy with preserved ovaries. Rather than study the variation in the magnitude and/or direction of the effect of menopausal experiences on employment among women who used HT and those who did not (ie, HT as an effect modifier) we considered HT as a mediator, ie, as a potential pathway through which menopausal experiences affect employment. Evidence for mediation can help to identify targeted interventions to support women in the workplace. However, we also observed the direct effect of premenopausal gynecological surgery on labor market exit, independent of HT use, suggesting that other pathways, such as health, may play more important roles as mediators.

### Strengths and limitations

The ELSA Life History interview provides invaluable insights into the complete work histories and comprehensive data on the menopause experiences of women in England born in the first half of the 20th century. It allowed us to explore the period surrounding the final menstruation or surgery for each woman, despite differences in the timing of these events. Furthermore, it enabled us to provide robust covariate adjustment across domains such as childhood adversity, health, accommodation, relationships, and fertility histories. Importantly, missing data are minimal and have been addressed through multiple imputation.

Several limitations need to be acknowledged. The information we used was collected retrospectively, which introduces the possibility of recall errors, especially as age increases. However, the reliability and validity of such data improve when event history calendars are used,^38^ as was the case with the ELSA Life History interview. Changes in job roles, such as moving from a full-time job with more responsibilities to a less demanding full-time job, could have occurred but would have been overlooked in this analysis. Due to sample size limitations, we combined economic activity statuses, such as part-time work and self-employment. However, these forms of work are conceptually similar in terms of less standard work arrangements, potentially offering more flexibility to manage menopause experiences. Our study’s observation period included time before the final menstruation or surgery, making it challenging to separate the effects of menopause or surgery from other factors, including work-related ones. This may also compromise the mediation analysis in this study, where the temporality of exposure, mediator, and outcome are essential. As menopause is a process, and surgeries often involve pretreatment burdens necessitating the surgical intervention, we deemed the period around the final menstruation or surgery more important for this study than using the final period as a cutoff. We categorized surgical menopause as either premenopausal bilateral oophorectomy or hysterectomy. When ovaries are preserved, hysterectomy does not lead to immediate menopause and women classified as having surgical menopause in our study may have varying experiences. We conducted an additional analysis excluding women who had a hysterectomy only before menopause. However, a larger sample size is needed to obtain more reliable evidence on the differences in their experiences. In this study, postmenopause HT did not account for formulation, dose, duration, or method of administration. Its use may have been influenced by the severity of complaints (eg, asymptomatic women may not require treatment), availability, accessibility, current knowledge and guidelines, publicity, and other factors. Measurement error in the mediator can contribute to bias in the mediation analysis and underestimate the indirect effects. Another limitation of the IOW mediation method is that the variance of estimates can be wider than that of traditional parametric mediation methods, making it more difficult to detect small indirect effects, as observed in our analysis.^28,29^ In line with current thinking in this area, our interpretation of results moves away from the traditional reliance on *P* values as threshold screening tools and binary confidence interval interpretation. Instead, the presented confidence intervals are intended to reflect plausible value ranges and inherent uncertainty.^39^ Lastly, the world of work has evolved over the years, and variations in the timing of reproductive events, including menopause, have been reported. These variations may potentially limit the generalization of findings across generations.

## CONCLUSIONS

Our study highlights that early and surgical menopause, including premenopausal hysterectomy, impact women’s labor market trajectory during the transition into menopause and suggests that HT within the early years of the final menstruation may help affected women remain employed. This study strengthens the case for further research in larger studies, which would provide more precise estimates, and advocates for workplace menopause policies that consider the diverse menopausal experiences of women, the timing and type of menopause.
